# Proprotein Convertase Subtilisin/Kexin Type 9 Inhibitors Use for Atherogenic Dyslipidemia in Solid Organ Transplant Patients

**DOI:** 10.3390/jcm11113247

**Published:** 2022-06-06

**Authors:** Gianluigi Cuomo, Giuseppe Cioffi, Anna Di Lorenzo, Francesca Paola Iannone, Giuseppe Cudemo, Anna Maria Iannicelli, Mario Pacileo, Antonello D’Andrea, Carlo Vigorito, Gabriella Iannuzzo, Francesco Giallauria

**Affiliations:** 1 Department of Translational Medical Sciences, “Federico II” University of Naples, Via S. Pansini 5, 80131 Naples, Italy; gianluigi.cuomo95@gmail.com (G.C.); gcioffi316@gmail.com (G.C.); dilorenzoanna2@gmail.com (A.D.L.); giuseppe.cudemo@unina.it (G.C.); annamaria.iannicelli@unina.it (A.M.I.); vigorito@unina.it (C.V.); 2 Department of Clinical Medicine and Surgery, “Federico II” University of Naples, Via S. Pansini 5, 80131 Naples, Italy; francesca.p.iannone@gmail.com (F.P.I.); gabriella.iannuzzo@unina.it (G.I.); 3 Unit of Cardiology and Intensive Care, Umberto I Hospital, 84014 Nocera Inferiore, Italy; pacmario@yahoo.it (M.P.); antonellodandrea@libero.it (A.D.)

**Keywords:** dyslipidemia, immunosuppressants, transplant, PCSK9, PCSK9 inhibitors, alirocumab, evolocumab

## Abstract

Dyslipidemia is a widespread risk factor in solid organ transplant patients, due to many reasons, such as the use of immunosuppressive drugs, with a consequent increase in cardiovascular diseases in this population. PCSK9 is an enzyme mainly known for its role in altering LDL levels, consequently increasing cardiovascular risk. Monoclonal antibody PCSK9 inhibitors demonstrated remarkable efficacy in the general population in reducing LDL cholesterol levels and preventing cardiovascular disease. In transplant patients, these drugs are still poorly used, despite having comparable efficacy to the general population and giving fewer drug interactions with immunosuppressants. Furthermore, there is enough evidence that PCSK9 also plays a role in other pathways, such as inflammation, which is particularly dangerous for graft survival. In this review, the current evidence on the function of PCSK9 and the use of its inhibitors will be discussed, particularly in transplant patients, in which they may provide additional benefits.

## 1. Introduction

Dyslipidemia is defined as the occurrence of abnormal plasma levels of any lipid and/or lipoprotein fraction. This condition is globally widespread, but there is higher prevalence (about 50%) in developed countries, such as in Europe and in North America, whereas in developing countries, the prevalence is about 25% [1]. Dyslipidemia, particularly high levels of serum low-density lipoprotein (LDL), is the main risk factor for the development of atherosclerosis and cardiovascular disease (CVD) [2], which caused about 523 million cases and 18.5 million deaths in 2019: Moreover, it is a relevant cause of disability, with about 194 million disability-adjusted life years (DALYs) estimated in the same year [3].

Transplant recipients are part of a particular population suffering from atherogenic dyslipidemia. In fact, lipid alterations have been found in 80% of kidney transplant recipients [4] and heart transplant recipients [5], and 50% of liver transplant recipients [6]. Consequently, this lipid elevation is associated with an increase in CVDs, which become the first cause of mortality in kidney and heart transplant recipients [7,8]. In addition, dyslipidemia is also associated with other complications occurring in these patients, such as cardiac allograft vasculopathy (CAV) in heart transplant recipients [9], and chronic allograft nephropathy (CAN) in kidney transplant recipients [10].

However, in these patients, the management of atherogenic dyslipidemia is challenging, since lipid elevation is caused by immunosuppressants (that cannot be withdrawn) [11,12] and due to the interactions between immunosuppressants and statins or other lipid-lowering drugs [13]. For this reason, often, statins and ezetimibe are used at a lower dosage, complicating LDL level reduction and CVD prevention [14].

In recent years, a therapy with Proprotein Convertase Subtilisin/Kexin type 9 (PCSK9) inhibitors is available for patients suffering from familial hypercholesterolemia or who are defined as very-high risk for the occurrence of cardiovascular events by the European Society of Cardiology guidelines on dyslipidemia [15,16]. They are human monoclonal antibodies which have proven to be safe and effective in reducing the incidence of major cardiac adverse events (MACE) in the general population [17,18,19].

This clinical success could be too simplistically attributed to the reduction in total cholesterol, LDL, and lipoporotein (a) (Lp(a)) levels alone: in fact, these drugs can also counteract the atherosclerotic process through other mechanisms, such as anti-inflammatory effects, plaque stabilization, anti-aggregation, and anticoagulant effects [20,21].

In transplant patients, due to the restrictive criteria for the prescription of PCSK9 inhibitors (PCSK9i), few cases have been reported with efficacy comparable to the general population [22]. Moreover, these drugs undergo a metabolism pattern different than immunosuppressants; therefore, there are no pharmacokinetic interferences [13,14].

In addition, elevation in PCSK9 levels due to the use of immunosuppressants has been shown [23,24], and this could be one more reason to use PCSK9i in this population.

This review aims at describing the role of the PCSK9 pathway in atherogenic dyslipidemia in transplant recipients, comparing the effectiveness of PCSK9i use in both the general population and in transplant patients. New emerging categories of patients that could benefit from the use of PCSK9i have been proposed.

## 2. Role of PCSK9 in Dyslipidemia and Atherosclerosis

PCSK9 is a proteolytic enzyme first discovered in neurons and involved in the apoptosis mechanism [25].

This protein has a ubiquitous distribution, but is predominantly found in the liver, small intestine, kidney, and muscular tissue [26]. In recent years, it has received much attention for its role in lipid metabolism.

The hepatocytes, where PCSK9 is primarily expressed, are the main drivers for LDL particle removal from blood, through endocytosis mediated by the LDL receptor (LDLR) expressed on these cells’ membrane [27]. PCSK9 target the epidermal growth factor-like repeat homology domain A (EGFA-like) located on the LDLR, so LDLR and LDL particles are enabled to separate, and the entire complex is directed to lysosomes for degradation, avoiding the receptor recycling on the cell surface, and reducing, in this way, the clearance of LDL particles from plasma [17,26].

Gain-of-function mutations of the PCSK9 gene have been shown to cause familial hypercholesterolemia (FH), enhancing the risk for atherosclerosis and CVDs [28,29]. Missense mutations’ gain-of-function in the same gene is linked to hypercholesterolemia, due to reduction in LDLR expression on the cell surface; in reverse, loss-of-function mutations cause hypocholesterolemia and lower LDL plasma levels until 40% [30].

Furthermore, PCSK9 is also related to the modulation of total cholesterol and VLDL levels, through overproduction of apolipoprotein B (ApoB) [31]. PCSK9 overexpression in hepatocytes increases VLDL production and circulating triglycerides levels [32,33], and mice without PCSK9 present reduced post-prandial triglycerides levels [34]. PCSK9 implication in VLDL regulation is also proven by the finding that mice with homozygosis deletion for the PCSK9 gene accumulate more visceral adipose tissue and have higher levels of VLDL receptor [35].

In addition to these effects on metabolism, PCSK9 also directly promotes the atherosclerotic process. In fact, lipid accumulation in the intima layer of the vessel is not the only factor responsible for atheroma formation, as other factors promote the initiation and progression of atherosclerosis. In particular, inflammatory cells play a main role: macrophages start the process of phagocytosing oxidized LDL particles and transforming into foam cells, which begins a local inflammatory reaction, with consequent release of cytokines that attract other monocytes [36]; in later stages, T lymphocytes recruited into the lesion release other cytokines, causing the accumulation of collagen and glycosaminoglycans, which enlarge the plaque [37]. PCSK9 has been shown to have a role in the inflammatory response: on the one hand, promoting monocytes recruitment in the atherosclerotic lesion [38], and, on the other hand, activating leukocytes in a pro-inflammatory state, with consequent release of cytokines, in particular IL-17, which is associated with the atherosclerotic process [39]. Furthermore, PCSK9 is also produced by endothelial cells, and as a consequence of reactive oxygen species (ROS) production, the expression of this enzyme is enhanced, causing the hyperexpression of adhesion molecules which promote monocytes adhesion, favoring plaque progression [40].

Moreover, PCSK9 has also been shown to play a role in hemostasis, and, therefore, in clot formation that causes CVD: on the one hand, it can promote CVD, also increasing platelet aggregation and, consequently, thrombus formation, binding the CD36 receptor present on the thrombocyte membrane [41]; on the other hand, it increases the concentration of coagulation factors both directly, as on fibrinogen [42], and indirectly through the action on LDLR, which influences the factor VIII levels [43,44].

CD36, as well as lecitin-like oxidized low-density lipoprotein receptor-1 (LOX-1), also act as receptors for oxidized LDL particles in macrophages, provoking their transformation in foam cells, and both are enhanced by PCSK9 in inflammatory status [42].

In addition, PCSK9 levels are associated with a higher plasma concentration of fibrinogen (r = 0.211, *p* = 0.002) and C reactive protein (CRP) (r = 0.153, *p* = 0.023) in patients with coronary artery disease (CAD) [45].

Therefore, PCSK9 is involved in several pathways promoting atherosclerosis, and for this reason, it has become a target for atherosclerotic dyslipidemia therapies, particularly in patients who have a very-high risk for CVD.

**Figure 1:**
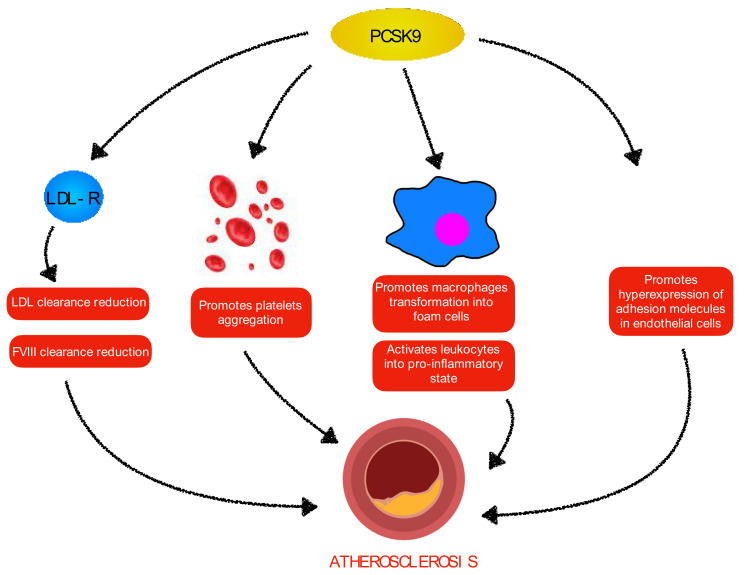
Mechanisms by which PCSK9 leads to atherosclerosis. FVIII: coagulation factor VIII; LDL: low-density lipoprotein; LDL-R: low-density lipoprotein receptor; PCSK9: proprotein convertase subtilisin/kexin type 9.

Figure 1 shows the different mechanisms by which PCSK9 promotes atherosclerosis.

## 3. PCSK9 Inhibitors Efficacy in Cardiovascular Diseases

The two PCSK9i used in clinical practice for optimizing LDL-lowering therapy are the human monoclonal antibodies, evolocumab and alirocumab. They are administered through a 2- or 4-week subcutaneous or intramuscular injection, and have been shown to decrease LDL plasma concentration and reduce ischemic cardiovascular events. Another PCSK9i, the humanized monoclonal antibody called bococizumab, has not been placed on the market because of its higher rate of side reactions and increased production of neutralizing antibodies [46].

Alirocumab and evolocumab have been shown in clinical trials to reduce LDL serum levels, on average, by 60%, so the latest European Society of Cardiology guidelines on dyslipidemia recommend their use (class IA) in combination with the maximum-tolerated dose of a statin and ezetimibe in secondary prevention patients, and in FH patients with another major risk factor for CVD who do not achieve the LDL levels target [15].

The Further Cardiovascular Outcomes Research with PCSK9 Inhibition in Subjects with Elevated Risk (FOURIER) trial investigated the efficacy of evolocumab in addition to statin therapy in reducing the incidence of MACE in 27,564 patients (75% were males; average age, 63 years) with CVD and LDL levels ≥ 70 mg/dL (median LDL levels, 92 mg/dL), who were randomized in an evolocumab group (*n* = 13,784) and a placebo group (*n* = 13,780). A mean reduction in LDL levels of 59% (from 92 mg/dL to 30 mg/dL, *p* < 0.001) and a reduction of primary MACE endpoints (HR 0.85; 95% CI: 0.79–0.92; *p* < 0.001) were observed in the treatment group [18]. In addition, prespecified analysis showed a doubling of MACE prevented with evolocumab over a longer observation time, suggesting the benefit of continuing aggressive lipid-lowering therapy [47].

In the ODYSSEY LONG TERM trial, alirocumab at 150 mg every 2 weeks showed a statistically significant reduction in LDL levels (−62%, *p* < 0.001) compared to placebo, and in a post hoc analysis, the reduction in MACE was also lower (HR 0.52; 95% CI: 0.31–0.90; *p* = 0.02) [48].

Moreover, in the ODYSSEY OUTCOMES trial, 18,924 patients who experienced an acute coronary syndrome in the previous 1–12 months and had LDL levels ≥ 70 mg/dL or ApoB level ≥ 80 mg/dL despite statin therapy were randomly assigned to alirocumab at a 75 mg subcutaneous dose every 2 week group (*n* = 9462) or a placebo group (*n* = 9462): alirocumab showed great results in terms of LDL levels reduction (about 60% lower than the placebo group), and a reduction in composite primary endpoints (HR 0.85; 95% CI: 0.78–0.93; *p* < 0.001), which were death from CAD, nonfatal myocardial infarction, ischemic stroke, or unstable angina requiring hospitalization [19].

Furthermore, for both evolocumab and alirocumab, there were no differences in the rate of serious adverse events, allergic reactions, and diabetes, but injection-site reactions were more common than with placebo (3.8% for alirocumab, and 2.1% for evolocumab) [18,19].

In addition, PCSK9i showed other effects. Unlike statins, these monoclonal antibodies are effective in reducing, by about 25–30%, the circulating levels of lipoprotein (a) (Lp(a)) [49], which is associated with a higher risk of CVD [50,51].

Moreover, PCSK9i can reduce the atherosclerotic plaque: the GLAGOV trial showed that the addition of evolocumab to statin therapy in patients with CAD decreases the total atheroma volume by—4.9 mm^3^, and stimulates a strong plaque regression compared to placebo (64.3% vs. 47.3%) [52].

Finally, PCSK9i efficacy in preventing CVD is also plausibly due to the pleiotropic effects on hemostasis [20]: in 21 patients with isolated hypercholesterolemia, these drugs showed a significant reduction in plasma levels of fibrinogen (*p* = 0.01), factor VII (*p* = 0.01), and plasminogen activator inhibitor-1 (PAI) (*p* = 0.001).

## 4. Atherogenic Dyslipidemia and Cardiovascular Diseases in Transplant Patients

Solid organ transplantation is a therapy which allows patients with end-stage organ disease to survive. Due to recent innovations in the field of transplantation, there has been a reduction in early complications, such as rejection and infections, which have long-been the major cause of mortality in these patients, and, consequently, the life expectancy of these patients has increased [53].

Nevertheless, other diseases now threaten the survival of these patients, such as CVDs, which have become the first causes of death in heart transplant patients [8] and kidney transplant patients [7], and the second causes of death in liver transplant patients [54,55]. It should be considered that kidney transplant recipients are particularly at risk for CVD because, due to end-stage chronic kidney disease, they are classified as having a very-high risk for CVD [56].

However, this increase is not solely due to the longer life expectancy, which entails the possibility of the appearance of diseases common to the general population. In fact, transplant recipients appear to be more exposed to risk factors for CVD. In particular, atherogenic dyslipidemia is present in about 80% of kidney transplant recipients [4], 80% of heart transplant recipients [5], and 70% of liver transplant recipients [57], compared to about 30–40% of the general population [58,59]. These variations are due to multiple factors, such as genetic [60,61,62] and lifestyle [63,64] factors; however, the main role is probably played by immunosuppressants, which exert pharmacodynamic and pharmacokinetic interferences on lipid and glycemic metabolism [11,65]: in fact, in the first months after transplantation, during which immunosuppressants are administered at a higher dose, higher total cholesterol serum levels were found [66].

Among immunosuppressants, the mammalian target of rapamycin (mTOR) inhibitors, sirolimus and everolimus, are strongly associated with an increase in triglycerides, VLDL, and LDL, because they inhibit LPL function; reduce catabolism of the apolipoproteins, ApoB100 and apoCIII; alter insulin secretion; and induce pancreatic β-cells apoptosis [67,68,69]; these alterations are probably responsible for the significantly higher incidence of cardiovascular disease shown in patients treated with mTOR inhibitors (ROR 1.95, 95% CI: 1.70–2.23) [70].

The calcineurin inhibitors, cyclosporine (CsA) and tacrolimus, reduce the activity of lipoproteinlipase (LPL) and hepatic lipase with an increase in hepatic lipogenesis and apolipoprotein CIII (apoCIII), increasing serum levels of total cholesterol, LDL, and triglycerides [71,72,73]. CsA also causes drug interactions with statins, making the achievement of the LDL target by lipid-lowering therapies difficult [13,14].

In addition to atherogenic dyslipidemia, with increased levels of LDL and triglycerides, these drugs are responsible for other risk factors promoting atherosclerosis, such as arterial hypertension and hyperglycemia [11,65,74]. In fact, 20–40% of patients who have undergone solid organ transplant develop post-transplant diabetes mellitus (PTDM) [75], which involves an increase in CVD and reduced survival [76]. Several predisposing factors have been investigated for PTDM [77,78,79,80], but this complication is mostly associated with the use of immunosuppressants, particularly glucocorticoids [81,82], calcineurin inhibitors [83,84], and mTOR inhibitors [85,86].

Moreover, some immunosuppressants cause arterial hypertension, which occurs in more than 50% of transplant recipients, and is a well-known risk factor for CVD [87]. Calcineurin inhibitors, and particularly CsA, are the principal cause of arterial hypertension in transplant patients, both by activating production of vasoconstrictor molecules and reducing the release of nitric oxide (NO) [88,89]: proving this, the discontinuation of these drugs often resolves hypertension [65].

A main role in initiation and progression of atherosclerotic plaque in transplant recipients is also played by inflammation, which is increased due to the immune reaction against donor organs [90,91]. For this reason, in heart transplant patients, there is a particular form of CAD, named CAV, which is caused by antibodies against donor antigens that activate T lymphocytes, and the consequent inflammatory reaction leads to endothelial proliferation and vessel occlusion [9]. CAV is responsible for about 10% of deaths in these patients, and is distinguished from the usual CAD for its pathognomonic lesions, which affect the intramuscular arteries and microvascular bed [92,93,94].

In kidney transplant recipients, the increased inflammatory state causes the formation of oxidized LDL particles [95], which are associated with CAN, which can lead to transplant failure [10,96,97].

In conclusion, CVDs result more frequently in transplant patients due to a greater exposure to risk factors, caused both by the inflammatory state of the transplant, and by the side effects of immunosuppressants.

**Figure 2:**
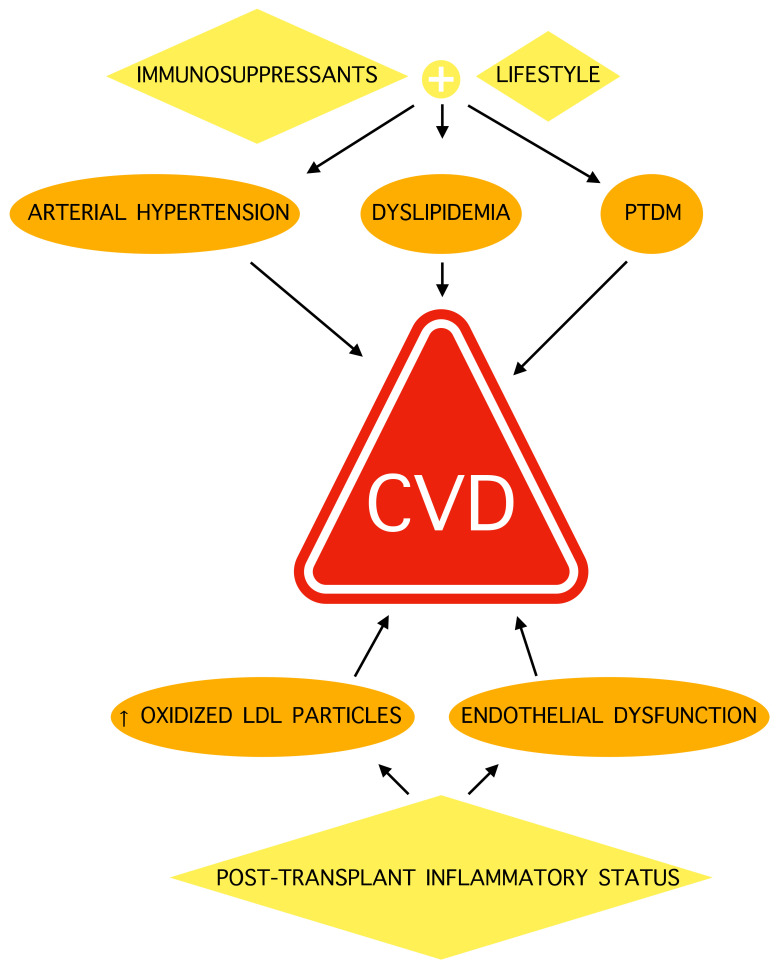
Cardiovascular disease mechanisms in transplant patients. CVD: cardiovascular disease; PTDM: post-transplant diabetes mellitus. LDL: low-density lipoprotein.

In Figure 2, the mechanisms of CVD in transplant recipients are summarized.

## 5. PCSK9 Inhibitors Use in Transplant Patients

The 2019 European Society of Cardiology guidelines on dyslipidemia consider transplant patients a special population that need attention [15].

However, as there is no risk category for transplant recipients, each patient should be assessed individually based on their clinical history. Kidney transplant patients are an exception, as end-stage chronic kidney disease places them in the very-high risk class [98]. Moreover, heart transplant patients should keep LDL serum levels < 100 mg/dL to avoid the occurrence of CAV [99].

Anyway, lipid alterations in these patients cause not only CVD, but are also associated with transplant failure, as in CAN [10].

In these patients, statin use is often used at a lower dosage due to drug interactions with immunosuppressants, and as a result, it is not sufficient to achieve LDL target levels [13,14].

PCSK9i have been shown to be effective in reducing LDL levels in the general population by about 60% if used alone, 75% if used with high-intensity statins, and 85% if ezetimibe is added [16,100].

Based on their pharmacodynamic and pharmacokinetic properties, these monoclonal antibodies should not create drug interactions with immunosuppressants, and do not act on the cytochrome system or other enzymes involved in their metabolism [13].

To date, less evidence is available on the use of these drugs in transplant patients, since the indications for their use are limited to patients in the secondary prevention for CVD, or with familial hypercholesterolemia [15,16].

The first case reports were carried out in heart transplant recipients, and showed encouraging data on LDL level reduction, calling for larger trials or the creation of registries to also evaluate the safety of these drugs in solid organ transplant patients [101,102].

More recently, Sammour et al. [103] investigated efficacy and safety of PCSK9i in 65 patients who underwent a heart transplant in last 10 years: a mean reduction of 58% in LDL circulating levels was found 3 months after therapy initiation (from 130 mg/dL to 55 mg/dL; *p* < 0.001), and this significant reduction was confirmed at last follow-up (median follow-up time, 1.6 years) [103]. A total of 72% of patients achieved an LDL level target of <70 mg/dL at first follow-up, and significant reductions in total cholesterol and triglycerides, and significant increases in HDL were shown [103]. In addition, in 33 patients, through coronary angiography with intravascular ultrasound (IVUS), no progression of atherosclerotic plaque was observed [103].

In regard to this action on coronary disease, in the next years, the EVOLVD trial will try to prove the reduction in CAV incidence in heart transplant patients treated with evolocumab [104].

In kidney transplant recipients, there is scarce evidence about PCSK9i use. A recent case report described the experience of a 54-year-old female patient who underwent kidney transplantation in 2011 with atherogenic dyslipidemia arising during everolimus and tacrolimus treatment [105]: one year after initiation of alirocumab, a significant decrease of 83.1 mg/dL in LDL circulating levels (*p* = 0.04) and of 94.9 mg/dL in total cholesterol (*p* = 0.03) were observed, while no side effects or drug interactions with immunosuppressants occurred, and kidney function remained stable [105].

Another report described the case of a male kidney transplant patient who experienced two episodes of respiratory infections, the first of which required hospitalization in the intensive care unit for 48 h, after the initiation of therapy with alirocumab [106]; however, these episodes did not recur after everolimus was replaced with azathioprine.

Warden et al. described the use of PCSK9i in a more heterogeneous population, including nine heart transplant patients, one kidney transplant patient, one liver transplant patient, and one lung transplant patient [22]: 6 months after treatment, a median decrease of 60% in LDL circulating levels was observed, and all patients achieved the target of <70 mg/dL. Moreover, there were no problems with immunosuppressant therapeutic ranges, and no transplant rejection occurred [22].

The possibility that PCSK9i may cause an immune-interfering effect in transplant patients should be taken into account, especially given the reasons that led to bococizumab withdrawal [46]. However, it must be considered that other types of monoclonal antibodies with immunosuppressive effects are already successfully used in transplant recipients, and currently, no immune-interfering effect has been found with the human monoclonal antibodies, alirocumab and evolocumab.

In reverse, there are some findings about PCSK9 in transplant patients, suggesting potential additional benefits that PCSK9i could have in this population.

For example, PCSK9 serum levels have been shown to increase 6 months after kidney transplantation [107]: although it can be hypothesized that this is due to the role of PCSK9 in inflammation [108], this does not seem plausible given that an inverse trend has been observed in inflammatory markers, such as leukocytes, IL-6, and CRP [107]. Authors hypothesized that this increase should be responsible for the higher incidence of atherogenic dyslipidemia in kidney transplant recipients and graft dysfunction, hoping for new research on the possibility that PCSK9i could promote longer renal graft survival [107].

Moreover, the administration of everolimus in renal and heart transplant patients demonstrated a statistically significant increase in PCSK9 levels [23,24], especially in patients with the mTORC1 rs2295080G polymorphism (*p* = 0.006) [24]: although it was not correlated with an increase in LDL levels, as the authors suggested, PCSK9 level elevation should represent an independent risk factor for the onset of CVD [109] or graft vasculopathy [23].

Finally, recent evidence showed that higher PCSK9 serum levels are associated with the development of PTDM, and this association is independent of statin use [110]. This association could be due to the regulatory role that PCSK9 has on the LDLR, which, in turn, appears to influence each other with the insulin receptor [111].

In addition, insulin resistance has been observed to cause an increase in hepatocyte PCSK9 transcription [112], so the increase in PCSK9 levels in patients with PTDM may be just a sign of insulin resistance from other causes. 

However, the role of PCSK9 in diabetes, especially with this type in transplant patients, is still unclear.

So, the alterations in PCSK9 levels in transplant recipients are due to several factors. The use of PCSK9i, in addition to the well-known effect on lipid metabolism and cardiovascular risk, may underline, in future studies, more benefit in transplant patients. The assessed and possible benefits of these drugs’ use in transplant recipients are summarized in Table 1.

## 6. Conclusions

Atherogenic dyslipidemia in solid transplant recipients is often more difficult to treat, due to the role of immunosuppressants, and often, many patients do not reach the LDL level goal, resulting in an increase in cardiovascular disease.

PCSK9i have been shown to be very effective and safe drugs even in these patients; in fact, they can be used together with immunosuppressants without causing drug interactions that could cause serious side effects or reduce graft survival.

Furthermore, post-transplantation status and the use of drugs such as everolimus have been shown not only to favor atherosclerosis and CVD incidence, but also increase PCSK9 levels to a greater extent, and this can cause well- or less-known consequences: in fact, in addition to causing an increase in plasma levels of LDL and probably favoring post-transplant diabetes mellitus, it remains to be clarified whether elevation in PCSK9 levels can influence graft survival.

The use of PCSK9i in this population should be further investigated, as in addition to improving dyslipidemia and cardiovascular outcomes, they could have additional benefits on transplant complications and graft survival.

## Figures and Tables

**Table 1:** Shown and possible PCSK9 inhibitor use benefits in transplant patients, CVD: cardiovascular disease; FVIII: coagulation factor VIII; LDL: low-density lipoprotein.

**BENEFITS SHOWED**	Reduction in LDL levels in patients already at the highest possible dose of statins, without drug interferences with immunosuppressants.
	Reduction in CVD incidence and atherosclerotic plaque progression.
**POSSIBLE BENEFITS**	Reduction in cardiac allograft vasculopathy incidence.
	Reduction in post-transplant diabetes mellitus incidence.
	Reduction in chronic allograft nephropathy incidence.
	Graft survival extension.
	Reduction in CVD through platelet aggregation inhibition.
	Reduction in CVD through FVIII clearance.

## Data Availability

Data sharing not applicable.
